# Nomogram for predicting risk of mild renal dysfunction among general residents from rural Northeast China

**DOI:** 10.2478/jtim-2023-0003

**Published:** 2024-07-27

**Authors:** Shasha Yu, Hongmei Yang, Bo Wang, Xiaofan Guo, Guangxiao Li, Yingxian Sun

**Affiliations:** Department of Cardiology, First Hospital of China Medical University, Shenyang 110001, Liaoning Province, China; Department of Clinical Epidemiology, Institute of Cardiovascular Diseases, First Hospital of China Medical University, Shenyang 110001, Liaoning Province, China

**Keywords:** mild renal dysfunction, estimated glomerular filtration rate, rural, prediction, epidemiology

## Abstract

**Background and objectives:**

Cumulative evidence confirms that mild renal dysfunction (MRD) is correlated with many cardiovascular risk factors and increases cardiovascular morbidity and mortality. The purpose of this study was to establish an effective nomogram for predicting the risk of MRD in the rural population of Northeast China.

**Methods:**

We analyzed the reports of 4944 subjects from the Northeast China Rural Cardiovascular Health Study (NCRCHS). All the participants completed the questionnaires, anthropometric measurements, and blood tests during the baseline study (2012–2013) and the follow-up study during 2015–2017 (an average of 4.6 years). The Chronic Kidney Disease Epidemiology (CKD-EPI) equation was used to calculate the estimated glomerular filtration rate (eGFR), and eGFR in the range of 60–90 mL/min/1.73m2 was defined as MRD.

**Results:**

The study revealed that a total of 889 subjects (18.0%) had MRD. Multivariate logistic analysis showed that annual income, abdominal obesity, hypertension, hyperglycemia, and frequent tea consumption were the independent risk factors (*P* < 0.05) for MRD. Thereafter, a nomogram with an area under the receiver operating characteristic curve (AUC) of 0.705 was constructed to accurately predict MRD. The calibration plot also showed an excellent consistency between the probability of prediction and observation.

**Conclusion:**

We constructed a nomogram based on epidemiological data, which could provide an individual prediction of MRD with good accuracy.

## Introduction

Mild renal dysfunction (MRD) is a growing concern worldwide, and there is increasing evidence that it may affect many cardiovascular diseases (CVDs) and cerebrovascular diseases. The Good Aging in Skåne (GÅS) cohort study confirmed that MRD was associated with impairment in learning and memory, language, complex attention, executive function, and global cognitive function, but not meta-memory.^[1]^ Park *et al*.^[2]^ reported that the probability of variations in the left ventricular (LV) geometry was higher in men than in women with MRD. The Framingham Heart study also inferred that individuals with mildly reduced estimated glomerular filtration rate (eGFR) had a higher probability of subclinical atherosclerosis, cross sectionally, and a greater risk of CVD and chronic kidney disease (CKD) progression.^[3]^ Similarly, a study has shown that MRD might be associated with a combined increase in ventricular systolic stiffness and arterial load in patients with known or suspected coronary artery disease (CAD), leading to an increased risk of heart failure.^[4]^ Therefore, it is necessary to find effective methods to predict MRD in order to reduce the risk of CVD.

There is a great variation in MRD prevalence between the developed and developing areas. Data from the Framingham Heart Study showed a high prevalence of mildly reduced eGFR (62%),^[3]^ which was remarkably higher than those observed in the studies conducted in developed countries like Australia (43%), the USA (52.1%), and Turkey (20%).^[5, 6, 7]^ Similarly, the prevalence of mildly reduced eGFR among urban populations in Bangladesh was 24%.^[8]^ A study conducted to estimate the prevalence of CKD in Cameroonians found that the subjects living in the urban areas had a significantly higher prevalence of MRD (15.1%) compared to those living in the rural areas (9.1%).^[9]^ In addition, Burkhalter *et al*.^[10]^ reported that the MRD prevalence in the rural regions of Haiti was 6.4%, which was lower than that in the urban areas. Previous studies also confirmed that a variety of metabolic disorders, like fasting insulin, hyperglycemia, hyperuricemia, and dyslipidemia, significantly increased the risk of mildly reduced eGFR.^[11, 12, 13]^ However, Onat *et al*.^[7]^ claimed that insulin resistance rather than metabolic disorders determined MRD, which was also supported by the National Health and Nutrition Examination Survey (NHANES).^[14]^ Furthermore, a community-based study conducted by Ji *et al*.^[15]^ in the urban areas of Chongqing, a large city in southwestern China, indicated that compared to the higher eGFR group, the waist circumference (WC), systolic blood pressure (SBP), fasting plasma glucose (FPG), and uric acid (UA) of the mildly reduced eGFR group were significantly increased. In addition, Hou *et al*.^[16]^ found that triglyceride (TG) levels are closely associated with a mild decline in the eGFR in the middle-aged and elderly Chinese population with normal serum lipid levels. Concerning the possible association between socioeconomic status and renal dysfunction, Shen *et al*.^[17]^ found that the prevalence of MRD was higher in subjects belonging to a farming background or with low level of education, whereas the income levels were irrelevant in the rural population of the North-Central Zhejiang province in eastern China.

Compared to the urban residents, the rural residents in China have relatively few medical resources and are also less likely to be concerned about their health.^[18]^ Furthermore, the New Cooperative Medical Scheme (NCMS) and the Urban Residents’ Basic Medical Insurance System (URBMI) have failed to improve the inequalities in health financing between the urban and rural residents.^[19]^ Our cross-sectional study showed that the awareness rates of hypertension, diabetes, and dyslipidemia were 17.1%, 3.9%, and 5.8%, respectively,^[20,21]^ and even less concern was expressed toward MRD. Our previous study, China Rural Hypertension Control Project, concluded that compared to intensive routine care, rural doctor-led interventions showed statistically significant improvements in blood pressure (BP) control among the rural population in China.^[22]^ Integrated analysis can, therefore, provide useful insights, and effective evaluation and prediction will further help the village doctors identify subjects at potential risk. However, thus far, no studies have been conducted on MRD in rural areas. The purpose of this study is to investigate the incidence and risk factors of MRD and make recommendations for its prevention based on the novel nomogram.

## Materials and methods

### Study population

**Figure 1:**
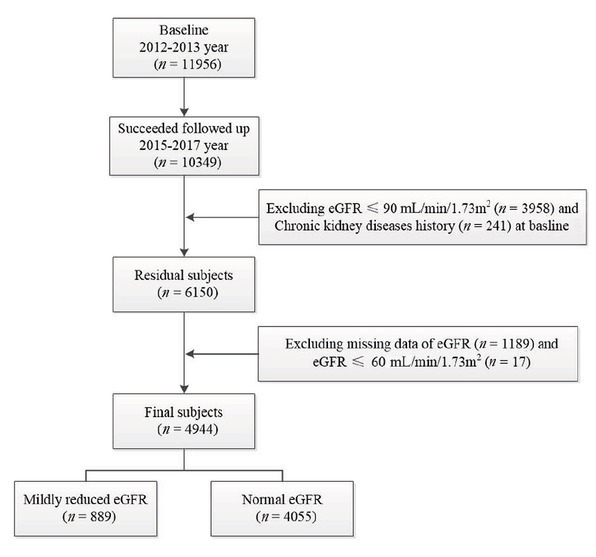
Flow chart of participants included and excluded in this study. eGFR: estimated glomerular filtration rate.

The Northeast China Rural Cardiovascular Health Study (NCRCHS) is a community-based cohort study conducted in the rural areas of Northeast China, and the design and inclusion criteria of this study have been described previously.^[21,23]^ Figure 1 depicts the detailed recruitment and selection process of the participants. A total of 11,956 participants completed the study, with an 85.3% response rate. This study was approved by the China Medical University’s Ethics Committee (Shenyang, China AF-SDP-07-1, 0-01). The participants were invited to participate in the follow-up studies in 2015 and 2017. The average duration of the follow-up period was 4.66 years. All the participants provided written informed consent for the study.

### Data collection and variables definition

Data regarding age, gender, race, marital status, physical activity, education, annual income, sleep duration, history of chronic diseases, smoking/alcohol consumption status, soybean consumption, and tea consumption were obtained from the standard questionnaire. Continuous variables including age, annual income, and sleep duration were then transformed into dichotomous variables with cutoff values of 50–65 years, 5000–20,000 CNY, and 7–9 h, respectively. CVD (angina, myocardial infarction, arrhythmia, and heart failure), cerebrovascular disease (cerebral hemorrhage, cerebral infarction, subarachnoid hemorrhage, transient ischemic attack), and CKD (nephritis, acute/chronic renal failure) were used as examples of chronic disease status.

BP was measured thrice (in 5-min intervals) using a standardized automatic electronic sphygmomanometer (HEM-907; Omron, Tokyo, Japan). Fasting blood samples were collected in the morning from the participants who had fasted for at least 12 h. Enzymatic analyses were performed on FPG, total cholesterol (TC), low-density lipoprotein cholesterol (LDL-C), high-density lipoprotein cholesterol (HDL-C), TG, serum creatinine, and other routine blood biochemical indexes. Additionally, the WC values of 90 cm in men and 80 cm in women were considered as abdominal obesity.^[24]^ Patients on antihypertensive drugs and with blood pressure > 130/85 mmHg were identified to have a history of hypertension. Additionally, elevated LDL-C, low HDL-C, and hypertriglycerides were identified as dyslipidemia. Hyperuricemia was defined as serum UA (SUA) level of ≥ 416 μmol/L in men and ≥ 357 μmol/L in women. A blood glucose level of ≥ 100 mg/dL or drug treatment for elevated glucose was defined as hyperglycemia. The CKD epidemiology (CKD-EPI) equation was used to calculate the eGFR.^[25]^ MRD was defined as eGFR of 60–90 mL/min/1.73m^2^.

### Statistical analysis

Statistical analysis was performed using Statistical Package for the Social Sciences (SPSS) version 22.0 statistical software (SPSS Inc., Chicago, IL, USA) and R statistical software (R Foundation for Statistical Computing, Vienna, Austria).^[26]^ Descriptive statistics were computed for all the variables, including continuous variables (reported as mean values and standard deviations) and category variables (reported as numbers and percentages). The *t*-test, analysis of variance (ANOVA), analysis of covariance (ANCOVA), nonparametric tests, and the two-test were used to assess differences between categories. Univariate logistic regression analysis was used to identify the risk factors for MRD. All the *P*-values were bilateral, and the risk factors with *P*-values < 0.05 in univariate analysis were included in the multivariate analysis. Multivariate logistic regression was used to identify independent risk factors, and a stepwise method was used to identify the combination of factors that best predicted MRD. Thereafter, a nomogram based on the multivariate logistic regression was developed for MRD.

## Results

### Population characteristics and the incidence ofMRD

A total of 4944 subjects were included in the analysis. Among these, 50.4% of the subjects were males, with an average age of 50.48 ± 8.29 years. Serum lipid, FPG, SUA, BP, and WC were measured at the baseline study. In the follow-up study, we identified 889 subjects who were diagnosed with MRD. The descriptive characteristics of the study subjects are shown in Table 1.

**Table 1:** Descriptive characteristics of study population (*N* = 4944)

Characteristics	Results
Age (years)	50.48 ± 8.29
Male	2491 (50.4)
Current smoking (yes)	1909 (38.6)
Current drinking (no)	1349 (27.3)
Ethnicity^a^ (Han)	4564 (92.3)
Marriage (yes)	4895 (99.0)
Regular exercise (yes)	794 (16.1)
Education status	
Primary school or below	2113 (42.7)
Middle school	2274 (46.0)
High school or above	557 (11.3)
Annual income (CNY/year)	
≤ 5000	473 (9.6)
> 5000 and ≤ 20,000	2767 (55.9)
> 20,000	1704 (34.5)
Sleep duration (hour/day)	
≤ 7	2261 (45.7)
> 7 and ≤ 8	1546 (31.2)
> 8 and ≤ 9	721 (14.6)
> 9	416 (8.4)
Chronic diseases^b^ (yes)	850 (17.2)
Waist circumference (cm)	82.00 ± 9.67
Systolic blood pressure (mmHg)	140.85 ± 22.75
Diastolic blood pressure (mmHg)	81.95 ± 11.46
LDL-C (mmol/L)	2.90 ± 0.81
HDL-C (mmol/L)	1.46 ± 0.40
Total cholesterol (mmol/L)	5.12 ± 1.03
Triglyceride (mmol/L)	1.54 ± 1.52
Fasting plasma glucose (mmol/L)	5.82 ± 1.66
Serum uric acid (mmol/L)	275.73 ± 79.14
Beans or Bean product frequency	
Rarely	1774 (35.9)
2–3 times/week	2514 (51.1)
≥ 4 times/week	636 (12.9)
Tea consumption frequency	
None	2754 (55.8)
Occasionally	1087 (22.0)
1–2 times/day	967 (19.6)
≥ 3 times/day	131 (2.7)
Mildly impaired function	889 (18.0)

Data are presented as mean ± SD or *n* (%). ^a^Others include some ethnic minorities in China, such as Mongol and Manchu. ^b^Chronic diseases include cardiovascular diseases (angina, myocardial infarction, arrhythmia, and heart failure), cerebrovascular diseases (cerebral hemorrhage, cerebral infarction, subarachnoid hemorrhage, transient ischemic attack), and chronic kidney diseases (nephritis, acute/chronic renal failure). CNY: China Yuan (1 CNY = 0.161 USD); LDL-C: low-density lipoprotein cholesterol; HDL-C: high-density lipoprotein cholesterol.

### Risk factors associated with MRD

A comparative analysis between subjects with and without MRD suggested that MRD was prevalent in subjects belonging to the following categories: older age, male, currently smoking, Han ethnicity, low level of education, relatively low annual income, history of chronic diseases, abdominal obesity, hypertension, hyperuricemia, and low daily tea consumption (*P* < 0.05). Multivariate logistic analysis identified higher annual income (5000–20,000 CNY/year: 0.74 [0.59, 0.95] and > 20,000 CNY/year: 0.67 [0.51, 0.87]), abdominal obesity (1.23 [1.04, 1.44]), hypertension (1.58 [1.31, 1.91]), hyperglycemia (0.59 [0.48, 0.72]), and tea consumption frequency (occasionally: 1.47 [1.21, 1.79]; one or two times/day: 2.18 [1.81, 2.64]; and ≥ 3 times/day: 2.15 [1.40, 3.24]) as the independent risk factors for MRD (Table 2).

**Table 2:** Univariate andmultivariate analyses for riskfactors of mildly reduced eGFR in ruralNortheast China

Variables	Group	Univariate			Multivariate	
			
		Mildly reduced	Non-mildly reduced	*P*-value	OR	95% CI
		eGFR (*n* = 889)	eGFR (*n* = 4055)			
Age (years)	35–49	284 (31.9)	2293 (56.5)	< 0.001	1 (reference)	
	50–64	471 (53.0)	1653 (40.8)		2.22	1.88, 2.63
	≥ 65	134 (15.1)	109 (2.7)		8.24	6.13, 11.12
Gender	Male	486 (54.7)	2005 (49.4)	0.003		
Current smoking	Yes	381 (42.9)	1528 (37.7)	0.002		
Current drinking	Yes	258 (29.0)	1091 (26.9)	0.108		
Ethnicity	Han	804 (90.4)	3760 (92.7)	0.014		
Marriage	Yes	883 (99.3)	4012 (82.0)	0.197		
Regular exercise	Yes	149 (16.8)	645 (15.9)	0.280		
Education status	Primary school or below	421 (47.4)	1692 (41.7)	0.007		
	Middle school	370 (41.6)	1904 (47.0)			
	High school or above	98 (11.0)	459 (11.3)			
Annual income	≤ 5000	134 (15.1)	339 (8.4)	< 0.001	1 (reference)	
(CNY/year)	> 5000 and ≤ 20,000	511 (57.5)	2254 (55.6)		0.74	0.59, 0.95
	﹥ 20,000	244 (27.4)	1460 (36.0)		0.67	0.51, 0.87
Sleep duration	≤ 7	401 (45.2)	1858 (45.9)	0.474		
(hour/day)	> 7 and ≤ 8	266 (30.0)	1278 (31.6)			
	> 8 and ≤ 9	138 (15.5)	582 (14.4)			
	> 9	83 (9.3)	332 (8.2)			
Chronic diseases^b^	Yes	193 (21.7)	657 (16.2)	< 0.001		
Abdominal obesity	≥ 90 cm for men	378 (42.9)	1529 (38.0)	0.004	1.23	1.04, 1.44
	≥ 80 cm for women					
Hypertension	Systolic ≥ 130 and/or	700 (79.4)	2602 (64.6)	< 0.001	1.58	1.31, 1.91
	diastolic ≥ 85 mm Hg^a^					
Dyslipidemia	Yes^b^	623 (70.1)	2885 (71.1)	0.275		
Hyperuricemia	≥ 416 μmol/L for men	112 (12.6)	242 (6.0)	< 0.001		
	≥ 357 μmol/L for women					
Hyperglycemia	≥ 100 mg/dL^c^	375 (42.2)	1753 (43.2)	0.295	0.59	0.48, 0.72
Beans or Bean product	Rarely	321 (36.2)	1453 (36.0)	0.598		
frequency	2–3 times/week	443 (49.9)	2071 (51.3)			
	≥ 4 times/week	123 (13.9)	513 (12.7)			
Tea consumption	None	384 (43.2)	2370 (58.5)	< 0.001	1 (reference)	
frequency	Occasionally	200 (22.5)	887 (21.9)		1.47	1.21, 1.79
	1–2 times/day	268 (30.2)	699 (17.3)		2.18	1.81, 2.64
	≥ 3 times/day	36 (4.1)	95 (2.3)		2.15	1.40, 3.24

Data are presented as n (%). ^a^Antihypertensive drug treatment in a patient with a history of hypertension is an alternate indicator. ^b^Include elevated LDL-C, low HDL-C, elevated total cholesterol, and hypertriglycerides. ^c^Drug treatment of elevated glucose is an alternate indicator. CNY: China Yuan (1 CNY = 0.161 USD); LDL-C: low-density lipoprotein cholesterol; HDL-C: high-density lipoprotein cholesterol; eGFR: estimated glomerular filtration rate; OR: odds ratio; CI: confidence interval.

### Development of the nomogram for MRD

**Figure 2:**
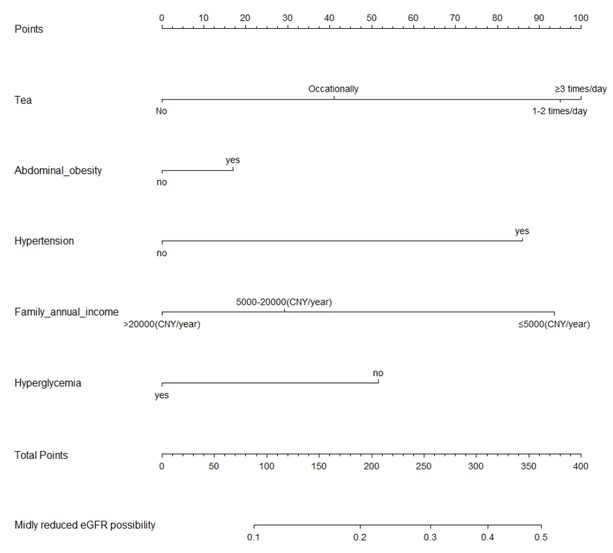
Nomogram for predicting risk of mildly reduced eGFR among rural Northeast Chinese. eGFR: estimated glomerular filtration rate.

**Figure 3:**
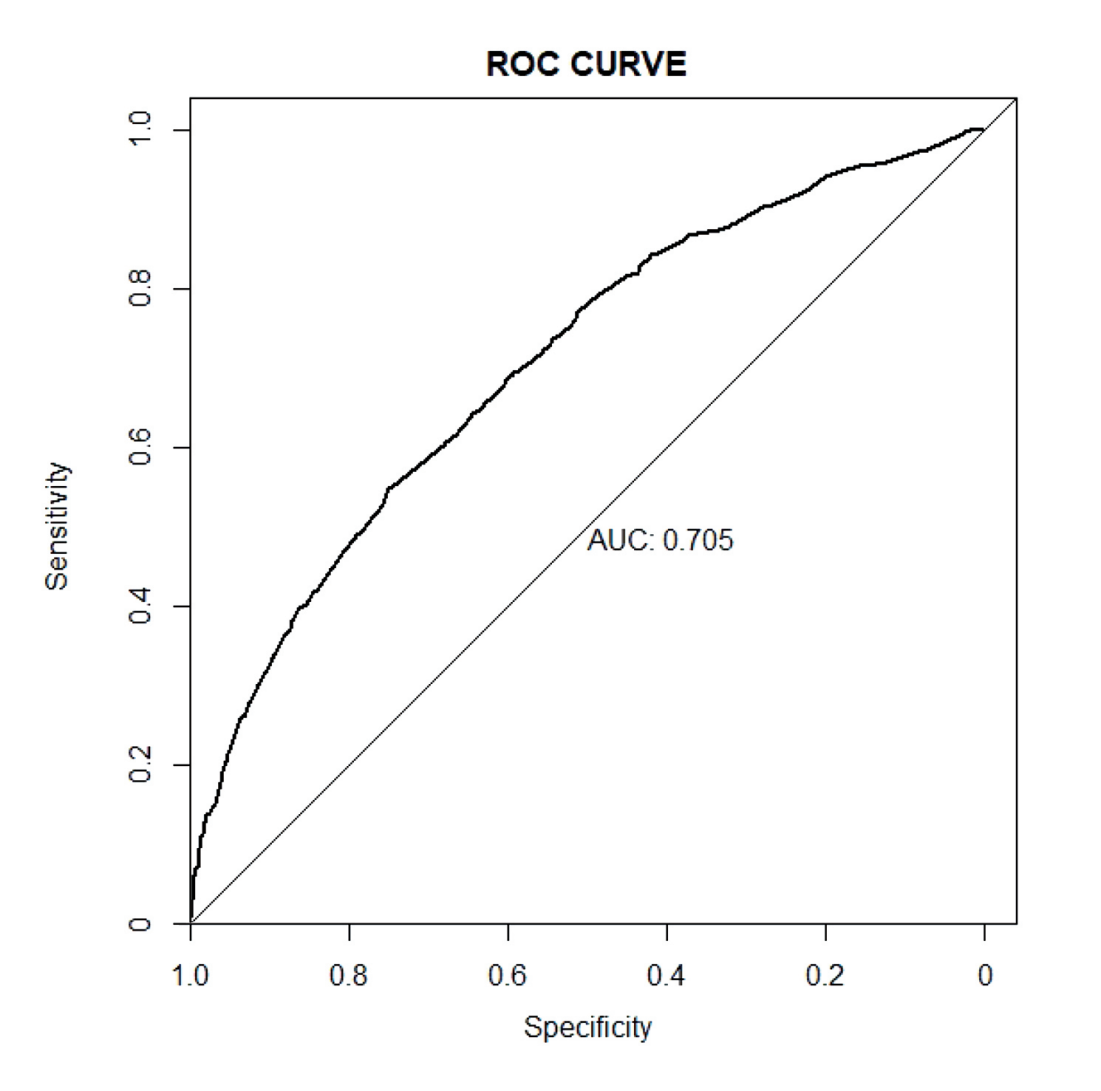
ROC analysis of the nomogram for mildly reduced eGFR among rural Northeast Chinese. AUC: area under curve; ROC: receiver operating characteristic; eGFR: estimated glomerular filtration rate.

**Figure 4:**
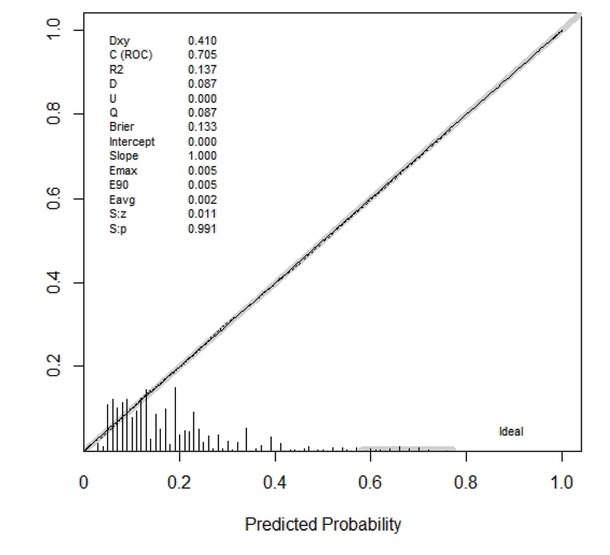
Calibration plot of the nomogram for mildly reduced eGFR among rural Northeast Chinese. Hosmer-Lemeshow test, *P*-value 0.232. eGFR: estimated glomerular filtration rate.

A nomogram incorporating the five significant risk factors was created (Figure 2), and the total scores were used to assess the risk for MRD. For instance, a rural resident, consuming tea one or two times/day, suffering from abdominal obesity and hypertension, earning 5000–20,000 CYN/year, and without hyperglycemia contributed 281.25 points (= 95 + 17.5 + 86.25 + 30 + 52.5) and had a 36.8% probability of developing MRD. The overall predictive accuracy of the nomogram, as measured by the bootstrap-corrected receiver operating characteristic (ROC) curve, was 0.705, indicating good discrimination (Figure 3). The calibration plot revealed that the nomogram is well calibrated and there is no significant difference between prediction probabilities and observation probability (Hosmer-Lemeshow test, *P* = 0.232) (Figure 4).

## Discussion

The primary finding of our study is that the proportion of MRD is relatively high in the Chinese rural general population. Therefore, effective prediction is needed for early diagnosis and prevention of renal damage. MRD was associated with annual income, abdominal

obesity, high BP, hyperglycemia, and tea consumption frequency. A new nomogram model was established to better evaluate the risk of MRD in the rural Northeast Chinese population.

Many previous studies have shown that decreased renal function is a predictor of hospitalization,^[27]^ cognitive impairment,^[28]^ and poor quality of life, often accompanied by an increased risk of cardiovascular events like arterial stiffness, coronary artery calcium, and myocardial hypertrophy.^[29]^ In addition, it has been an important issue in the management of CVD and cerebrovascular diseases. According to some previous studies, the prevalence of MRD varies by 2.8%–15.1%;^[30,31]^ however, in our study, a relatively higher cumulative incidence was observed, with 889 (18.0%) out of 4944 subjects experiencing MRD after 4.6 years of follow-up. The relative rates of hypertension (51.1%), diabetes (6.5% previously diagnosed; 8.7% undiagnosed), and dyslipidemia (36.9%) among the rural residents were higher than those recorded in the previous studies and could be responsible for the increased incidence of MRD during the follow-up.^[21, 32, 33]^ Hypertension, glucose levels, and lipid metabolism disorders have been estimated to increase the risk of MRD.^[34, 35, 36, 37]^ Furthermore, studies have linked an increase in eGFR to a steady decline in cardiovascular morbidity and mortality in subjects with MRD, suggesting an inverse relationship between eGFR and cardiovascular risk.^[38,39]^ Therefore, it is particularly important to assess the risk factors associated with MRD and make recommendations for its prevention.

Multifactorial etiologies were associated with MRD. In the current study, univariate and multivariate analyses identified six independent risk factors for MRD. Previous studies reported that the prevalence of dyslipidemia and diastolic BP variability was higher in subjects with MRD than in subjects with normal renal function.^[15,40]^ As previously claimed, elevated TG (odds ratio [OR]: 1.25), decreased HDL-C (OR: 1.23), and obesity (OR: 1.22) were associated with increased risk of mildly reduced eGFR, while elevated FPG (OR: 0.64) was associated with lower risk of mildly reduced eGFR.^[41]^ Our results support that abdominal obesity, hypertension, and hyperglycemia are strongly associated with MRD, and that abdominal obesity is an independent risk factor for MRD, while hyperglycemia is negatively associated with MRD. Many pathophysiological pathways (*e.g*., chronic inflammation, elevated oxidative stress, and hyperinsulinemia) or a set of risk factors (*e.g*., insulin resistance, hypertension, and dyslipidemia) may explain the possible association between obesity and renal damage.^[42]^ Our results are consistent with those of Hu *et al*.^[41]^ who claimed that hyperglycemia was associated with a relatively lower risk of MRD, which is inconsistent with several previous studies.^[43,44]^ This is possibly attributed to the hyperfiltration due to elevated FPG and glycated hemoglobin (HbA1c) levels resulting in a temporary increase in eGFR.^[41]^ Another possibility is the relatively short follow-up duration, since the results can vary with time. Therefore, large prospective studies with relatively longer follow-up durations should be conducted to explore the possible association between hyperglycemia and mildly reduced eGFR.

Previous studies have shown that the rates of elevated diastolic blood pressure were significantly higher in subjects with mildly reduced eGFR than in subjects with normal eGFR.^[15,40]^ In addition, hypertension has been proven to be a well-established risk factor for the progression of CKDs.^[14,45]^ As the economy grows, the prevalence of metabolic disorders, such as obesity, hypertension, and hyperglycemia, is increasing rapidly in both urban and rural areas.^[20,46,47]^ However, the rates of awareness, treatment, and control are significantly lower among the rural Chinese population compared to the urban population.^[48,49]^ Additionally, the management of diabetes and hypertension in the Chinese population is far from satisfactory. According to a Chinese national survey, only 32.2% of patients with diabetes mellitus (DM) received treatment and only 49.2% of the treated patients had < 7.0% HbA1c level.^[50]^ In addition, the rural Chinese population had significantly lower rates of awareness (4.6%) and treatment (3.7%). According to the China Hypertension Survey (2012–2015), 23.2% of Chinese adults suffer from hypertension, 46.9% are aware of their hypertension diagnosis, 40.7% take prescribed antihypertensive medications, and 15.3% have controlled hypertension.^[51]^ Similarly, our previous study on the rural Chinese population revealed that the awareness, treatment, and control rates for new cases of hypertension were 29.9%, 19.5%, and 1.5%, respectively.^[52]^ Low rates of awareness, treatment, and control in rural areas can lead to serious complications and cardiovascular mortality.

Interestingly, frequent tea consumption also increased the risk of MRD in the rural population. Tea consumption has been prevalent in Asia for centuries, especially in Japan and China, where it is the most consumed beverage after water. Tea is a rich source of pharmacologically active molecules and has many health benefits.^[53]^ Cumulative evidence from cell culture and animal studies confirms the beneficial effects of tea polyphenols in a wide range of pathological diseases, including cancer, diabetes, and CVDs.^[54,55]^ In addition, tea consumption prevents increased creatinine clearance and has antiproteinuric effects.^[56,57]^ However, most of the previous studies on tea consumption were conducted in cell cultures or experimental animal models. One recent cross-sectional study of the general Japanese population found that consumption of green tea may increase the risk of mildly reduced eGFR in Japanese men with NADH dehydrogenase subunit-2 237 leucine/methionine (ND2-237 Leu/Met) polymorphism.^[58]^ In contrast, a large population-based study on the older Chinese population showed no clear evidence between renal function and tea consumption.^[59]^ Therefore, it might be one possible explanation for the inconsistent results on the relationship between tea consumption and renal function, and further studies are required to confirm this association.

Nomograms are graphical tools or models that are used to estimate the probability of an event and accurately predict the outcomes for individual subjects. We developed a nomogram with six predictors that displayed good accuracy and discrimination in predicting the risk of MRD, with a corrected area under the ROC curve (AUC) of 0.705. Using this model, rural clinicians can accurately estimate the risk of MRD in rural subjects and tailor treatment to those who require close follow-up. To our knowledge, our study is the first to assess the risk of MRD in rural subjects. However, this study has a few limitations: (1) we did not assess the gene polymorphism among rural subjects, which could have explained the association between tea consumption and MRD; (2) the eGFR calculation was based on a single blood test, which can introduce bias; (3) although our analyses were adjusted for many possible confounding factors, residual confounding attributed to the measurement error in the assessment of confounding factors, unmeasured factors, such as family history of DM, hypertension, CVD, and other chronic diseases and antihyperglycemic, antihypertensive, and antihyperlipidemic therapeutic agents, cannot be excluded; (4) we only assessed the renal function by the eGFR equation; therefore, further studies are required to identify the risk factors for MRD by considering the other important indicators such as proteinuria, microalbuminuria, or urine microalbumin– creatinine ratio; and (5) even though we excluded the subjects with MRD at the baseline study, we did not adjust some factors that could possibly affect eGFR, such as the medicine consumed.

## Conclusion

The results of our study indicate that MRD is prevalent among rural residents in Northeast China. Additionally, MRD is more prevalent in rural subjects with relatively higher annual income, abdominal obesity, hypertension, and higher tea consumption frequency. We have constructed a novel nomogram that is a good predictor of MRD and can serve as a guide for epidemiological screening.

## References

[1] Månsson T, Overton M, Pihlsgård M, Elmståhl S (2019). Impaired kidney function is associated with lower cognitive function in the elder general population. Results from the Good Aging in Skåne (GÅS) cohort study. BMC Geriatr.

[2] Park SK, Jung JY, Kang JG, Chung PW, Ryoo JH (2019). Mildly Decreased Renal Function and Its Relation to Left Ventricular Geometry Change. Circ J.

[3] Ataklte F, Song RJ, Upadhyay A, Musa Yola I, Vasan RS, Xanthakis V (2021). Association of Mildly Reduced Kidney Function With Cardiovascular Disease: The Framingham Heart Study. J Am Heart Assoc.

[4] Fukuta H, Ohte N, Wakami K, Asada K, Goto T, Mukai S (2011). Reduced renal function is associated with combined increases in ventricular-systolic stiffness and arterial load in patients undergoing cardiac catheterization for coronary artery disease. Heart Vessels.

[5] Pani A, Bragg-Gresham J, Masala M, Piras D, Atzeni A, Pilia MG (2014). Prevalence of CKD and its relationship to eGFR-related genetic loci and clinical risk factors in the SardiNIA study cohort. J Am Soc Nephrol.

[6] Coresh J, Selvin E, Stevens LA, Manzi J, Kusek JW, Eggers P (2007). Prevalence of chronic kidney disease in the United States. JAMA.

[7] Onat A, Hergenç G, Uyarel H, Ozhan H, Esen AM, Karabulut A (2007). Association between mild renal dysfunction and insulin resistance or metabolic syndrome in a random nondiabetic population sample. Kidney Blood Press Res.

[8] Das SK, Afsana SM, Elahi SB, Chisti MJ, Das J, Mamun AA (2019). Renal insufficiency among urban populations in Bangladesh: A decade of laboratory-based observations. PLoS One.

[9] Kaze FF, Meto DT, Halle MP, Ngogang J, Kengne AP (2015). Prevalence and determinants of chronic kidney disease in rural and urban Cameroonians: a cross-sectional study. BMC Nephrol.

[10] Burkhalter F, Sannon H, Mayr M, Dickenmann M, Ernst S (2014). Prevalence and risk factors for chronic kidney disease in a rural region of Haiti. Swiss Med Wkly.

[11] Wang C, Liang K, Zhang X, Li C, Yang W, Ma Z (2014). Metabolic abnormalities, but not obesity, contribute to the mildly reduced eGFR in middle-aged and elderly Chinese. Int Urol Nephrol.

[12] Toyama T, Furuichi K, Shimizu M, Hara A, Iwata Y, Sakai N (2015). Relationship between Serum Uric Acid Levels and Chronic Kidney Disease in a Japanese Cohort with Normal or Mildly Reduced Kidney Function. PLoS One.

[13] Qiu Y, Zhao Q, Gu Y, Wang N, Yu Y, Wang R (2019). Association of Metabolic Syndrome and Its Components with Decreased Estimated Glomerular Filtration Rate in Adults. Ann Nutr Metab.

[14] Chen J, Muntner P, Hamm LL, Fonseca V, Batuman V, Whelton PK (2003). Insulin resistance and risk of chronic kidney disease in nondiabetic US adults. J Am Soc Nephrol.

[15] Ji B, Zhang S, Gong L, Wang Z, Ren W, Li Q (2013). The risk factors of mild decline in estimated glomerular filtration rate in a community-based population. Clin Biochem.

[16] Hou X, Wang C, Zhang X, Zhao X, Wang Y, Li C (2014). Triglyceride levels are closely associated with mild declines in estimated glomerular filtration rates in middle-aged and elderly Chinese with normal serum lipid levels. PLoS One.

[17] Shen Q, Jin W, Ji S, Chen X, Zhao X, Behera TR (2019). The association between socioeconomic status and prevalence of chronic kidney disease: A cross-sectional study among rural residents in eastern China. Medicine (Baltimore).

[18] Sun Y, Gregersen H, Yuan W (2017). Chinese health care system and clinical epidemiology Clin Epidemiol.

[19] Zhu K, Zhang L, Yuan S, Zhang X, Zhang Z (2017). Health financing and integration of urban and rural residents’ basic medical insurance systems in China. Int J Equity Health.

[20] Li R, Li W, Lun Z, Zhang H, Sun Z, Kanu JS (2016). Prevalence of metabolic syndrome in Mainland China: a meta-analysis of published studies. BMC Public Health.

[21] Li Z, Guo X, Zheng L, Yang H, Sun Y (2015). Grim status of hypertension in rural China: results from Northeast China Rural Cardiovascular Health Study 2013. J Am Soc Hypertens.

[22] Sun Y, Mu J, Wang DW, Ouyang N, Xing L, Guo X (2022). A village doctor-led multifaceted intervention for blood pressure control in rural China: an open, cluster randomised trial. Lancet.

[23] Yu S, Guo X, Yang H, Zheng L, Sun Y (2014). An update on the prevalence of metabolic syndrome and its associated factors in rural northeast China. BMC Public Health.

[24] Alberti KG, Eckel RH, Grundy SM, Zimmet PZ, Cleeman JI, Donato KA (2009). Harmonizing the metabolic syndrome: a joint interim statement of the International Diabetes Federation Task Force on Epidemiology and Prevention; National Heart, Lung, and Blood Institute; American Heart Association; World Heart Federation; International Atherosclerosis Society; and International Association for the Study of Obesity. Circulation.

[25] Levey AS, Stevens LA, Schmid CH, Zhang YL, Castro AF, Feldman HI (2009). A new equation to estimate glomerular filtration rate. Ann Intern Med.

[26] R Core Team. R (2020). A language and environmentfor statistical computing.

[27] Gansevoort RT, Correa-Rotter R, Hemmelgarn BR, Jafar TH, Heerspink HJ, Mann JF (2013). Chronic kidney disease and cardiovascular risk: epidemiology, mechanisms, and prevention. Lancet.

[28] Etgen T, Chonchol M, Förstl H, Sander D (2012). Chronic kidney disease and cognitive impairment: a systematic review and meta-analysis. Am J Nephrol.

[29] Chin HJ, Song YR, Lee JJ, Lee SB, Kim KW, Na KY (2008). Moderately decreased renal function negatively affects the health-related quality of life among the elderly Korean population: a population-based study. Nephrol Dial Transplant.

[30] Hill NR, Fatoba ST, Oke JL, Hirst JA, O’Callaghan CA, Lasserson DS (2016). Global Prevalence of Chronic Kidney Disease - A Systematic Review and Meta-Analysis. PLoS One.

[31] Lv JC, Zhang LX (2019). Prevalence and Disease Burden of Chronic Kidney Disease. Adv Exp Med Biol.

[32] Shi WR, Wang HY, Chen S, Guo XF, Li Z, Sun YX (2018). Estimate of prevalent diabetes from cardiometabolic index in general Chinese population: a community-based study. Lipids Health Dis.

[33] Yu S, Sun Z, Zheng L, Guo X, Yang H, Sun Y (2015). Prevalence of Diabetes and Impaired Fasting Glucose in Hypertensive Adults in Rural China: Far from Leveling-Off. Int J Environ Res Public Health.

[34] Cortese F, Cecere A, Maria Cortese A, Andriani A, Truncellito L, Valente F (2020). Vascular, cardiac and renal target organ damage associated to arterial hypertension: which noninvasive tools for detection?. J Hum Hypertens.

[35] Mennuni S, Rubattu S, Pierelli G, Tocci G, Fofi C, Volpe M (2014). Hypertension and kidneys: unraveling complex molecular mechanisms underlying hypertensive renal damage. J Hum Hypertens.

[36] Amorim RG, Guedes GDS, Vasconcelos SML, Santos JCF (2019). Kidney Disease in Diabetes Mellitus: Cross-Linking between Hyperglycemia, Redox Imbalance and Inflammation. Arq Bras Cardiol.

[37] Lin PH, Duann P (2020). Dyslipidemia in Kidney Disorders: Perspectives on Mitochondria Homeostasis and Therapeutic Opportunities. Front Physiol.

[38] Van Biesen W, De Bacquer D, Verbeke F, Delanghe J, Lameire N, Vanholder R (2007). The glomerular filtration rate in an apparently healthy population and its relation with cardiovascular mortality during 10 years. Eur Heart J.

[39] Lewis JR, Lim W, Dhaliwal SS, Zhu K, Lim EM, Thompson PL (2012). Estimated glomerular filtration rate as an independent predictor of atherosclerotic vascular disease in older women. BMC Nephrol.

[40] Diaz KM, Feairheller DL, Sturgeon KM, Veerabhadrappa P, Williamson ST, Crabbe DL (2011). Increased nitric oxide and attenuated diastolic blood pressure variability in african americans with mildly impaired renal function. Int J Hypertens.

[41] Hu W, Wu XJ, Ni YJ, Hao HR, Yu WN, Zhou HW (2017). Metabolic syndrome is independently associated with a mildly reduced estimated glomerular filtration rate: a cross-sectional study. BMC Nephrol.

[42] Lakkis JI, Weir MR (2018). Obesity and Kidney Disease. Prog Cardiovasc Dis.

[43] Sun Y, Wang C, Yang W (2015). Fasting blood glucose, but not 2-h postload blood glucose or HbA1c, is associated with mild decline in estimated glomerular filtration rate in healthy Chinese. Int Urol Nephrol.

[44] Hu Y, Shi LX, Zhang Q, Peng NC (2019). Increased Risk of Chronic Kidney Diseases in Patients with Metabolic Syndrome: A 3-year Prospective Cohort Study. Curr Med Sci.

[45] Klag MJ, Whelton PK, Randall BL, Neaton JD, Brancati FL, Ford CE (1996). Blood pressure and end-stage renal disease in men. N Engl J Med.

[46] Yu S, Yang H, Guo X, Zheng L, Sun Y (2017). Metabolic syndrome and depressive symptoms among rural Northeast general population in China. BMC Public Health.

[47] He Y, Li Y, Bai G, Zhang J, Fang Y, Zhao L (2019). Prevalence of metabolic syndrome and individual metabolic abnormalities in China, 2002-2012. Asia Pac J Clin Nutr.

[48] Bundy JD, He J (2016). Hypertension and Related Cardiovascular Disease Burden in China. Ann Glob Health.

[49] Lu J, Lu Y, Wang X, Li X, Linderman GC, Wu C (2017). Prevalence, awareness, treatment, and control of hypertension in China: data from 1·7 million adults in a population-based screening study (China PEACE Million Persons Project). Lancet.

[50] Wang L, Gao P, Zhang M, Huang Z, Zhang D, Deng Q (2017). Prevalence and Ethnic Pattern of Diabetes and Prediabetes in China in 2013. JAMA.

[51] Wang Z, Chen Z, Zhang L, Wang X, Hao G, Zhang Z (2018). Status of Hypertension in China: Results From the China Hypertension Survey, 2012-2015. Circulation.

[52] Sun Z, Zheng L, Detrano R, Zhang X, Xu C, Li J (2010). Incidence and predictors of hypertension among rural Chinese adults: results from Liaoning province. Ann Fam Med.

[53] Hartley L, Flowers N, Holmes J, Clarke A, Stranges S, Hooper L (2013). Green and black tea for the primary prevention of cardiovascular disease. Cochrane Database Syst Rev.

[54] Khan N, Mukhtar H (2018). Tea Polyphenols in Promotion of Human Health. Nutrients.

[55] Ohishi T, Goto S, Monira P, Isemura M, Nakamura Y (2016). Anti-inflammatory Action of Green Tea. Antiinflamm Antiallergy Agents Med Chem.

[56] Fiorino P, Evangelista FS, Santos F, Motter Magri FM, Delorenzi JC, Ginoza M (2012). The effects of green tea consumption on cardiometabolic alterations induced by experimental diabetes. Exp Diabetes Res.

[57] Shin BC, Kwon YE, Chung JH, Kim HL (2012). The antiproteinuric effects of green tea extract on acute cyclosporine-induced nephrotoxicity in rats. Transplant Proc.

[58] Kokaze A, Ishikawa M, Matsunaga N, Karita K, Yoshida M, Ohtsu T (2013). Unexpected combined effects of NADH dehydrogenase subunit-2 237 Leu/Met polymorphism and green tea consumption on renal function in male Japanese health check-up examinees: a cross-sectional study. J Negat Results Biomed.

[59] van Hasselt TJ, Pickles O, Midgley-Hunt A, Jiang CQ, Zhang WS, Cheng KK (2014). Effects of tea consumption on renal function in a metropolitan Chinese population: the Guangzhou biobank cohort study. J Ren Nutr.

